# Spatial Patterns and Drivers of Angiosperm Sexual Systems in China Differ Between Woody and Herbaceous Species

**DOI:** 10.3389/fpls.2020.01222

**Published:** 2020-08-11

**Authors:** Yunyun Wang, Tong Lyu, Ao Luo, Yaoqi Li, Yunpeng Liu, Robert P. Freckleton, Shuguang Liu, Zhiheng Wang

**Affiliations:** ^1^National Engineering Laboratory for Applied Technology of Forestry & Ecology in Southern China, and College of Life Science and Technology, Central South University of Forest and Technology, Changsha, China; ^2^Institute of Ecology and Key Laboratory for Earth Surface Processes of the Ministry of Education, College of Urban and Environmental Sciences, Peking University, Beijing, China; ^3^School of Urban Planning and Design, Shenzhen Graduate School, Peking University, Shenzhen, China; ^4^Department of Animal and Plant Sciences, University of Sheffield, Sheffield, United Kingdom

**Keywords:** angiosperms, sexual systems, growth form, climate change, geographical pattern, macro evo-devo, plant height, China

## Abstract

Plant sexual systems play an important role in the evolution of angiosperm diversity. However, large-scale patterns in the frequencies of sexual systems (i.e. dioecy, monoecy, and hermaphroditism) and their drivers for species with different growth forms remain poorly known. Here, using a newly compiled database on the sexual systems and distributions of 19780 angiosperm species in China, we map the large-scale geographical patterns in frequencies of the sexual systems of woody and herbaceous species separately. We use these data to test the following two hypotheses: (1) the prevalence of sexual systems differs between woody and herbaceous assemblies because woody plants have taller canopies and are found in warm and humid climates; (2) the relative contributions of different drivers (specifically climate, evolutionary age, and mature plant height) to these patterns differ between woody and herbaceous species. We show that geographical patterns in proportions of different sexual systems (especially dioecy) differ between woody and herbaceous species. Geographical variations in sexual systems of woody species were influenced by climate, evolutionary age and plant height. In contrast, these have only weakly significant effects on the patterns of sexual systems of herbaceous species. We suggest that differences between species with woody and herbaceous growth forms in terms of biogeographic patterns of sexual systems, and their drivers, may reflect their differences in physiological and ecological adaptions, as well as the coevolution of sexual system with vegetative traits in response to environmental changes.

## Introduction

The sexual systems of plant species play a significant role in the evolution of angiosperm diversity (Charlesworth and Wright, 2001; Nazareno et al., 2013; Käfer et al., 2014; Sabath et al., 2016; Rosche et al., 2018), variations in reproductive strategies (Bawa, 1980), and population and community dynamics in response to climate changes (Queenborough et al., 2009; Wang et al., 2018). Understanding the spatial distribution of plant sexual systems at large scales is vital for understanding the functional biogeography of reproductive traits. Previous studies have shown that the geographical distributions of angiosperm sexual systems may ultimately reflect coevolution with vegetative traits that are tied closely to sexual systems (i.e. growth form, Vamosi et al., 2003; Moeller et al., 2017; Wang et al., 2020). Nonetheless, the determinants of geographical patterns of sexual systems remain controversial.

The evolution of plant sexual systems has been widely hypothesized to be closely linked to the evolution of plant growth forms (e.g. woody vs. herbaceous growth forms, Bawa et al., 1985; Renner and Ricklefs, 1995; Vamosi et al., 2003; Vamosi and Vamosi, 2004; Mitchell and Diggle, 2005; Soza et al., 2012). Generally, dioecy is significantly more frequent in woody flowering plant species with long lifespan than in other growth forms (Renner and Ricklefs, 1995; Sakai et al., 1995; Vamosi et al., 2003; Ward et al., 2005). In contrast, hermaphroditism is more common in herbaceous species with short lifespan (Senarath, 2008; Moeller et al., 2017). This is likely because obligately outcrossing dioecious species need a long lifespan to find mates, reproduce and complete their life cycle (Morgan et al., 1997; Aarssen, 2000). In contrast, hermaphrodites, being able to self-pollinate, are likely to accumulate more genetic load and suffer from an increased turnover rate (Klekowski and Godfrey, 1989; Chen and Li, 2008). Moreover, growth form may affect relationships between traits and the environment (Fitzjohn et al., 2014). For example, based on more than 88,400 species and six plant traits, Šímová et al. (2018) have found significant differences in trait-climate correlations between woody and herbaceous species. However, the biogeographical distributions of sexual systems of different growth forms across large-scale environmental gradients remain poorly understood.

Plant sexual systems and sexual reproduction are sensitive to climate variations (Hedhly et al., 2009; Eckert et al., 2010). Recent studies indicate that reductions in precipitation can change the allocation of resources to male and female functions within individual plants (Hultine et al., 2016), which further influences the expression and composition of plant sexual system in local floras. For example, dioecy is more common in humid areas, especially in rainforest trees (Lloyd, 1980; Sakai et al., 1995; Friedman and Barrett, 2009; Vary et al., 2011), although aridity has been shown to have contributed to the evolutionary transition from hermaphroditism to dioecy (Ashman, 2006). Climate warming can also affect the composition of sexual systems in local floras by causing a mismatch between the timing of flowering and pollinator abundance/presence, which may lead to declines of plants populations (Etterson and Mazer, 2016). Moreover, different growth forms have contrasting climate preferences. Warm and humid climates are more suitable for woody plants (Wang et al., 2011), while herbaceous plants often have broader climate adaptations and hence have widespread distributions (Curtis and Bradley, 2016). Therefore, the influences of climate on sexual system compositions may differ between woody and herbaceous species. However, the role of climate in driving spatial variations in sexual system compositions of woody and herbaceous species remains poorly understood (Gamble et al., 2018).

Evolutionary history might be expected to influence the distribution of sexual systems (Obbard et al., 2006; Barrett, 2013; Käfer et al., 2014). Self-pollinated populations likely accumulate harmful mutants, which may increase their extinction rate (Etterson and Mazer, 2016). Therefore, self-pollinated species may be more common in temperate environments with more short-lived species and younger flora than in tropical environments. In contrast, dioecy (obligate outcrossing) can reduce the expression of recessive deleterious mutations, which tends to reduce their extinction rate (Obbard et al., 2006). Therefore, dioecy may be more frequent in regions with older floras. These findings suggest that the composition of sexual systems within communities is likely associated with the evolutionary ages of species in a region. Additionally, woody and herbaceous species differ significantly in their evolutionary rates (Laroche et al., 1997; Kay et al., 2006). Woody species have longer reproductive cycles and lifespans, and tend to accumulate genetic changes more slowly than herbaceous species. Differences in evolutionary rates may lead to differences in the speed of climate-niche evolution between these two growth forms. For example, using more than 5,000 plant species, Smith and Beaulieu (2009) found that woody plants adapted to new climates at a rate two to ten times slower than herbs over the course of their evolution. Therefore, exploring the differences between the effects of evolutionary time on frequencies of sexual systems of woody and herbaceous species would improve our understanding of the evolutionary mechanisms underlying large-scale patterns in sexual system composition.

Here, using data on sexual systems and spatial distributions of 19,780 angiosperm species across China, we compare the geographical patterns in plant sexual system compositions between woody and herbaceous species, and explore the ecological and evolutionary determinants of these patterns. Specifically, we test the following hypotheses: (1) the geographical patterns in sexual system composition differ between woody and herbaceous species; (2) the relative contributions of different drivers (specifically climate, evolutionary age, and mature plant height) to these patterns differ between woody and herbaceous species. Dioecious species are more common in humid areas and in floras with older and more woody species, whereas hermaphroditic species are more common in temperate arid areas of northwestern China and in floras with younger and more herbaceous species.

## Materials and Methods

### Sexual System

We compiled a database on the sexual systems of angiosperms in China using published sources: *Flora of China* (Wu et al., 1994-2013), *Flora Republicae Popularis Sinicae* (126 issues of 80 volumes), *Seeds of Woody Plants in China* (Chen and Huang, 2001), and efloras (http://efloras.org/). The Tree of Sex (Ashman et al., 2014), TYR Plant Trait Database (www.try-db.org; Kattge et al., 2011), Botanical Information and Ecology Network (BIEN, Enquist et al., 2016, http://bien.nceas.ucsb.edu/bien/biendata/bien-3/) and journal publications (Sabath et al., 2016; Goldberg et al., 2017) were used to check and supplement the sexual system information in the database. For species with information of sexual systems from multiple sources, conflicting records were checked and removed. In total, we compiled data of sexual systems for 19,780 species from 2,506 genera and 262 families in China (**Table 1A**).

**Table 1:** The correlations between the geographical patterns in proportions of sexual systems among different growth forms.

Sexual System	All-Wood	All-Herb	Wood-Herb
Hermaphroditism	0.535***	0.702**	0.304*
Dioecy	0.172ns	0.636*	−0.068ns
Monoecy	0.593*	0.888***	0.382**

All, all species; Wood, woody species; Herb, herbaceous species. The significance was estimated using the Dutilleul’s modified t test, which accounts for the inflation of significance due to spatial autocorrelation (Package “SpatialPack”; Dutilleul et al., 1993).

Significance codes: ***P < 0.001, **P < 0.01, *P < 0.05, ‘.’ P < 0.1. The text is bolded when the correlation is significant.

We classified all species into three categories according to their sexual systems following Cardoso et al. (2018): dioecy (separate male and female individuals), monoecy (having pistils and stamens on separate flowers of the same plant), and hermaphrodites (having both male and female sex organs on the same flower). The category of dioecy includes the *androdioecious*, *gynodioecious*, and *polygamodioecious* species, while monoecy includes the *monoecious*, *andromonoecious*, and *gynomonoecious* species. Considering that a few species vary intraspecifically in their sexual systems (e.g., Dorken et al., 2017) in response to local abiotic or biotic conditions (e.g., climate variables or pollinator densities; Barrett and Harder, 2017), we excluded such species from the final dataset used in this study.

### Species Distribution Data

Data on the distributions of plant species were compiled from all national, provincial and local floras, including *Flora of China* (both Chinese and English versions), *Higher Plants of China*, *The Atlas of Woody Plants in China* (Fang et al., 2011; see Wang et al., 2009 for more information), etc. From these resources, we compiled only species distribution records at county-level or finer scales. Species distributions were further supplemented with data from National Specimen Information Infrastructure (NSII, http://www.nsii.org.cn/), and screened. NSII included over fifteen million specimen records that are published recently. In total, our dataset consists the distributions of 19,780 Chinese plant species with data for sexual systems.

Species distributions were rasterized to a grid with a spatial resolution of 100 × 100 km to eliminate the potential bias of area on subsequent analyses. We removed the grid cells with less than 50% of their area along the country borders and coastal areas. In all, 949 grid cells were included in the analyses reported below.

### Growth Form

Data on the growth forms of all angiosperm species in China were compiled from the *Flora of China* (Wu et al., 1994-2013) and *Flora Reipublicae Popularis Bulgaricae* (Kuzmanov and Kožuharov, 1968). All species were categorized into two growth forms: “woody” and “herbaceous”. Woody species included trees, shrubs and woody lianas, whereas “herbaceous” species included those recorded as herbs, herbaceous lianas, and subshrubs. Species with information on both sexual systems and geographical distributions included 10,200 woody species, 9,479 herbaceous species, and 101 undefined.

### Climate Data

Climate can influence flower morphology through its influences on reproductive performance (e.g., pollen production) during the flowering period (Zhang, 2006), and hence could influence sexual systems of plants. Moreover, the reproductive (gametophytic) phase in flowering plants is often highly sensitive to temperature or water stresses. Thus we selected four variables to evaluate the influences of climate, namely, mean temperature of coldest quarter (MTCQ), mean precipitation of warmest quarter (MPWQ), actual evapotranspiration (AET), and aridity index (AI). AET reflects the influences of both water availability and environmental energy, and is significantly associated with ecosystem primary productivity. AET was calculated using the Thornthwaite equation (Thornthwaite and Hare, 1955) from the average monthly temperature and precipitation that were obtained from the WorldClim database (Hijmans et al., 2005). AI reflects the level of aridity and was calculated as the ratio of mean annual precipitation to annual potential evapotranspiration. Higher AI values represent lower aridity. The average and the full range of values for each climatic variable within each grid cell were estimated with the zonal statistics tool in ArcGIS 10.0.

### Genus Age

**Figure 1:**
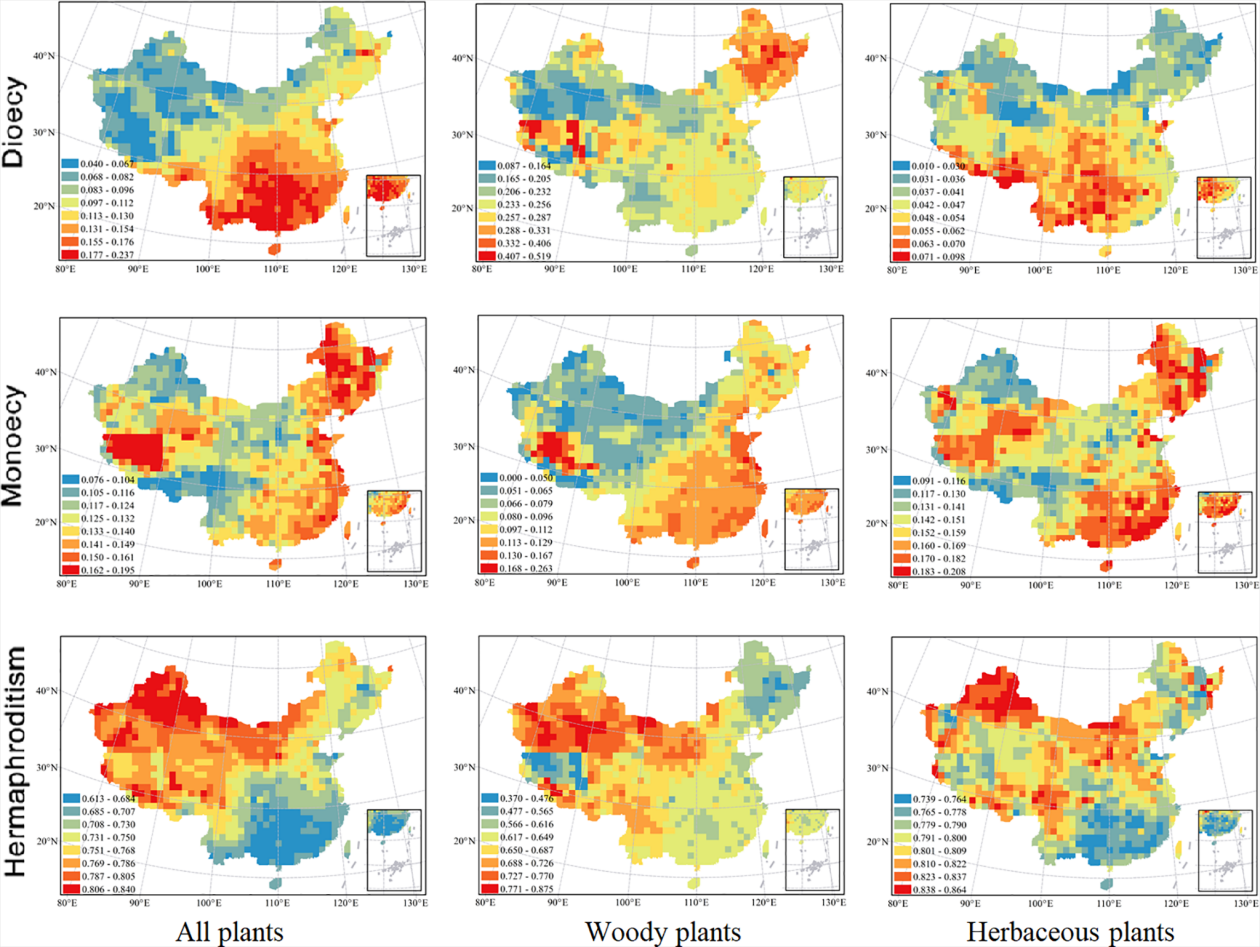
Spatial patterns in proportions of angiosperm species with different sexual systems at 100 × 100 km scale. From top down, the three rows represent the proportions of species with dioecy, monoecy, and hermaphroditism respectively. From left to right, the three columns represent all species, woody species and herbaceous species respectively. Grid cells without data are shown in white. The patterns in the proportions of sexual systems for woody species were updated from Wang et al., 2020.

To evaluate the effect of evolutionary time (i.e., genus age) on biogeographical patterns in sexual system compositions, we used the average genus age derived from three phylogenies including Zanne et al. (2014), Lu et al. (2018), and Smith and Brown (2018). This is because (1) different dating methods may lead to different estimations of genus ages; and (2) incomplete phylogenies tend to overestimate the ages of some genera when their close relatives are missed from the phylogenies, and none of these phylogenies is complete at the genus level. For comparison, we also repeated all analyses using the genus ages of each phylogeny separately. It is noteworthy that the geographical patterns in mean genus age per grid cell estimated using the genus ages derived from each phylogeny were consistent with each other (**Figure 1A**). Moreover, all major findings based on these four age estimations were also highly consistent. Therefore, we included the results based on the average genus ages across the three phylogenies in the main text, and those based on the genus ages derived from each phylogeny in the supplementary materials for comparison.

### Mature Plant Height

Plant mature height and longevity of plant species are expected to be positively associated (Marbà et al., 2007; Moles and Leishman, 2008; Moles et al., 2009). Here we used mature height as a proxy for longevity to test the association between sexual system and longevity. Mature height of plant species was obtained from *Flora of China* (http://frps.eflora.cn/, accessed in November 2013; http://www.efloras.org/flora_page.aspx?flora_id=2, accessed in February 2014). As lower and upper limits of mature height are normally reported for most species in floras, we used the average of lower and upper limits to represent the mature height of species. Species without erect stems (e.g., woody lianas, scandent shrubs, climbers or epiphytes) were excluded from our analyses following Moles et al. (2009). For each growth-form, we averaged the mature height across species within each grid cell to evaluate the effect of plant height on the biogeographical patterns of sexual system compositions (proportions of the three sexual systems per grid cell).

## Statistical Analyses

First, based on the data of sexual systems, growth forms, and distributions of all species, we calculated the proportions of species with different sexual systems within each grid cell for all species together and for each growth form separately. We used Pearson correlation coefficients to evaluate the similarity between the geographical patterns in the proportions of different sexual systems of woody and herbaceous species (e.g. woody dioecy vs. herbaceous dioecy, woody monoecy vs. herbaceous monoecy, woody hermaphroditism vs. herbaceous hermaphroditism) and used Dutilleul’s t test to evaluate the significance of these correlations due the influences of spatial autocorrelation on significance tests (using R package of “SpatialPack”; Dutilleul et al., 1993).

We used generalized linear models (GLMs) with quasi-Poisson residuals to evaluate the explanatory power of each predictor on the proportions of sexual systems per grid cell and this was conducted separately for each of the three species groups (i.e. overall, woody, and herbaceous). The climate variable (i.e., MTCQ, MPWQ, AET, and AI), genus age, and plant height were used as predictors and the proportions of each sexual systems per grid cell were used as response variables. The explanatory power of each variable was estimated as the adjusted R^2^_adj_ (%) of the GLM model. Modified t tests were used to correct for the effect of spatial autocorrelation on *p* values (Clifford et al., 1989).

To evaluate the influences of growth forms on the relationships between the proportion of sexual systems and different predictors, we first built spatial linear models (SLM) for the combined dataset of sexual system for both growth forms together using spatial simultaneous autoregressive error (SAR) models. SLMs could account for the effects of residual spatial autocorrelation on the significance tests of regression slopes (Kissling and Carl, 2008). The climate variables (i.e., AET, AI, MTCQ, and MPWQ), genus age, plant height, growth form (i.e., woody vs. herbaceous), and interaction terms between growth form and other predictors were included as the explanatory variables. All the predictors were standardized before fitting the models. Similarly, we also conducted SLMs for each growth form separately. Moran’s I was estimated for the residuals of SLMs, and indicated no spatial autocorrelations.

All analyses were performed in R 3.4.3 (Core Team R, 2017).

## Results

### Patterns in the Proportions of Sexual Systems for Different Growth Forms

**Figure 2:**
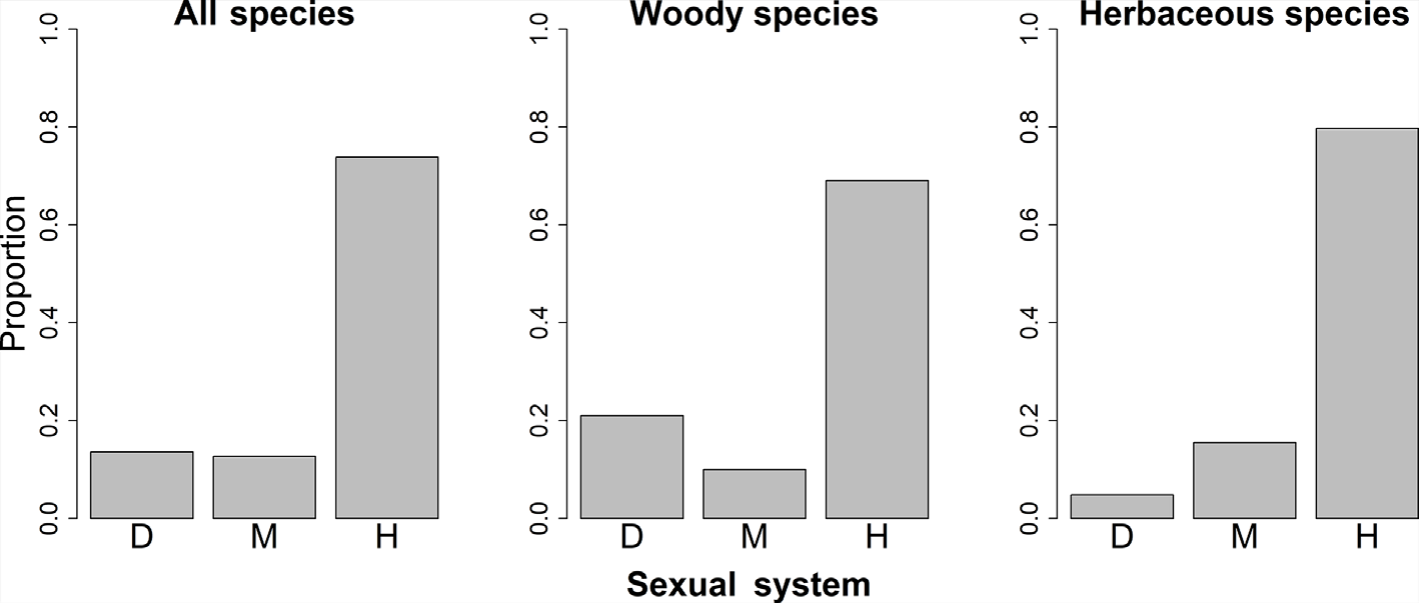
Proportions of species with different sexual systems among different growth forms in China. D, Dioecy; M, monoecy; H, hermaphroditism.

The spatial patterns in the proportions of hermaphroditic and monoecious species per grid cell were consistent between woody and herbaceous growth forms (r = 0.304 and p < 0.05 for proportion of hermaphroditic species; 0.382 and p < 0.05 for proportion of monoecious species; **Table 1**). For all species and both growth forms, the proportion of hermaphroditic species per grid cell was higher in northwest China than in other regions, while the proportion of monoecious species was higher in eastern China (**Figure 1**). In contrast, spatial patterns in the proportion of dioecious species were not significantly correlated between woody and herbaceous growth forms (*P* > 0.05; **Table 1**). The woody dioecious species were the most frequent in northeast China and medium in southern China, while herbaceous dioecious species were the most frequent in southwest China (**Figure 1**). Growth form was the strongest predictor for the proportions of all three sexual systems, and the effects of growth form on the proportions of sexual systems were higher than the other predictors by one order of magnitude (**Tables 2**, **3**, and **A2**; **Figure 2**).

**Table 2:** The slopes of growth forms (woody vs. herbaceous), climate, genus age, plant height, and their interaction on proportions of sexual systems (i.e. dioecy, monoecy, and hermaphroditism) evaluated using spatial linear models (SLMs) with simultaneous autoregressive errors (SAR).

Variables	Dioecy	Monoecy	Hermaphroditism
Growth Form	**0.169**	**−0.180**	−0.0192
Height	−0.0403	**0.112**	−0.0473
AET	**0.0101**	**0.00622**	−0.00657
AI	**0.00673**	0.00100	−0.00174
MTCQ	**0.00937**	**−0.00933**	−0.000256
MPWQ	**−0.0135**	−0.0000633	0.000311
Genus Age	0.00334	−0.00214	0.000283
Growth Form: AET	−0.0000394	−0.00167	0.000299
Growth Form: AI	−**0.0142**	−**0.0103**	**0.0244**
Growth Form: MTCQ	−**0.0333**	**0.0145**	**0.0205**
Growth Form: MPWQ	**0.0282**	**0.0195**	−**0.0475**
Growth Form: age	**0.0585**	**0.0266**	−**0.0831**
Growth Form: height	**0.0507**	−**0.113**	0.0423
R^2^	0.922	0.723	0.829

All continuous predictors were standardized prior analysis. The regression coefficients (slopes) with p < 0.05 is shown in bold. AET, actual evapotranspiration; AI, aridity index; MPWQ, mean precipitation of warmest quarter; MTCQ, mean temperature of coldest quarter; Age, evolutionary age. The genus age was calculated using the average genus age extracted from all three phylogenies including Zanne et al. (2014), Lu et al. (2018), Smith and Brown (2018).

**Table 3:** The slopes and significance of different predictors on proportions of sexual systems evaluated using spatial linear models (SLMs) with simultaneous autoregressive errors (SAR).

Variable	All			Herb			Wood		
	Herma.	Dioecy	Monoecy	Herma.	Dioecy	Monoecy	Herma.	Dioecy	Monoecy
AET	**−0.0173**	**0.0120**	**0.00866**	**−0.00707**	**0.00258**	**0.00446**	**−0.0183**	**0.0137**	0.00442
AI	**−0.00219**	**0.00740**	**−0.00410**	**−0.00250**	**0.00206**	0.000435	**0.0126**	**−**0.00434	**−0.00840**
MTCQ	**0.00738**	0.00455	**−0.0112**	**−**0.000611	**0.00260**	**−**0.00193	**0.0187**	**−0.0249**	**0.00619**
MPWQ	0.00130	**−0.0154**	0.00807	0.00138	**−**0.000430	**−**0.000962	**−0.0279**	0.00875	**0.0195**
Age	**−0.00518**	**0.00994**	**−0.00585**	0.000157	**0.00428**	**−0.00444**	**−0.0378**	**0.0270**	**0.0108**
Height	**−0.0228**	**0.0221**	**0.00500**	**−0.00429**	**−0.00174**	**0.00598**	**−−0.00588**	**0.00885**	**-0.00301**
R^2^	0.831	0.906	0.206	0.320	0.474	0.217	0.770	0.582	0.499

The regression coefficients (slopes) with p < 0.05 is shown in bold. Here genus age was average genus of the three phylogenies including Zanne et al. (2014), Lu et al. (2018), and Smith and Brown (2018).

### Effects of Climate on Distributions of Proportions of Sexual Systems

The proportions of all three sexual systems were relatively weakly related to climate for herbaceous species than for both all species and woody species (**Table 3**). The growth form–climate interaction terms were significant in almost all cases (**Table 2**). This indicates that the relationship between proportions of sexual systems and particular climate variables differed significantly between woody and herbaceous assemblages.

The effects of climate on proportions of sexual systems were significantly greater for woody species than for herbaceous species. For woody species, the proportion of hermaphroditism decreased with AET and MPWQ but increased with AI and MTCQ, while the proportion of dioecious showed almost opposite trends (**Table 3**). For herbaceous species, all three sexual systems were weakly correlated with climate (R^2^ ranged from 0.217 to 0.474 in **Table 3**).

### Effects of Genus Age on Geographical Distributions of Sexual Systems

Genus age was consistently a significant predictor of sexual system frequencies for woody species, but had only weak effect on herbaceous species. For woody species, the proportion of hermaphroditic was negatively correlated with genus age, while the proportion of dioecious species and monoecious species were positively correlated with genus age (all P < 0.05, **Table 3**). The explanatory power of genus age was much stronger on the proportions of dioecious and hermaphroditic species than on monoecious species (**Table 3**).

### Effects of Mature Plant Height on the Geographical Frequency of Sexual Systems

Mature plant height per grid cell was a significant predictor for geographical patterns in the proportions of almost all sexual systems examined here (**Tables 2** and **3**), but its effect was much stronger for woody species than for herbaceous species (**Tables 3** and **A2**). For both growth forms, the proportion of hermaphroditic species decreased. For woody species, the proportion of dioecious species increased but the monoecious decreased with plant height per grid cell, whereas herbaceous species showed opposite trend (**Table 3**).

## Discussion

Because different sexual systems are associated with variation of plant growth form, there are large-differences in the distributions of plant sexual systems between contrasting woody and herbaceous species. Compared with Wang et al. (2020), we advanced our understanding of the spatial distribution of plant sexual systems and the determinants underlying these patterns, especially between growth forms. Growth form significantly differed the relationships between geographic patterns in the proportions of all three sexual systems with different biotic and abiotic factors. Climate, evolutionary age, and mature plant height were all significantly correlates of the distributions of sexual systems of woody species than herbaceous ones. These results are partially consistent with the existing evidence that species diversity of woody plants is more closely related with family age and climate than that of herbaceous plants (Oberle et al., 2009; Hawkins et al., 2011).

### Effect of Plant Height on Proportions of Sexual Systems

Plant height was a significant predictor of the proportions of the three sexual systems in almost all cases, which partly confirmed previous hypotheses about the coevolution between sexual systems and growth forms (Renner and Ricklefs, 1995; Vamosi et al., 2003). Both plant height and sexual systems could reflect the adaptation and evolutionary trend of plant life-history strategies to a certain extent, and they also tend to coevolve (Renner, 2014; Wang et al., 2020). Owing to limited mating opportunities, selection has tended to favor dioecy in long-lived woody species which are tall (Renner and Ricklefs, 1995; Obbard et al., 2006). Taller species with height dimorphism and greater dispersal investment may also benefit dioecious species (e.g. *Salicaceae*) by improving the efficiency in pollen and seed dispersal, which further promote the maintenance and continuation of species populations (Pickup and Barrett, 2012; Thomson et al., 2018). In contrast, hermaphroditism tends to be associated with short-lifespan species with low height because hermaphroditic species can self-pollinate and thus tend to accumulate much more deleterious mutations (Etterson and Mazer, 2016), and which may have higher mortality and extinction risks (Obbard et al., 2006; Cheptou, 2019).

The explanatory power of plant height on the frequency of sexual systems was stronger for woody than for herbaceous species. This may be likely because woody and herbaceous plant species have different adaptive strategies in resource allocation between reproductive and vegetative tissues (Abrahamson and Gadgil, 1973; Bonser and Aarssen, 2003). Woody species may have more resources for the investment to sexual systems and reproduction (Porth and El-Kassaby, 2014), while herbaceous species (e.g. the annual *Mercurialis annua*) tend to exhibit short life-cycles, fast rates of population but limited resource accumulation (Lanfear et al., 2013).

### The Effects of Climate on the Proportions of Sexual Systems

The proportion of hermaphroditic species for all species combined was high in areas with high AI or MTCQ, consistent with Wang et al. (2020). The proportion of dioecious species among all species combined (e.g. *Anaphalis*) was higher in southern China than in other regions. This finding supports the hypothesis that dioecy is more common in tropical and subtropical floras where climate is warm and humid (Freeman et al., 1976; Bawa, 1980; Bawa et al., 1985; Sakai et al., 1995; Vary et al., 2011). In contrast, the proportion of woody dioecious species (e.g. *Rhamnus* and *Acer*) is higher in Northeast China with relatively higher AET and MPWQ compared to other regions in China. These results reveal that the differences in requirements for hydrothermal conditions between woody and herbaceous growth forms may influence their geographical patterns in proportions of sexual systems, consistent with our hypothesis. Generally, woody species often have deep roots, and herbaceous species have relatively shallow roots. Therefore, herbaceous species have lower efficiencies in soil water usage and are more sensitive to reduced water supply compared with woody species (Franco, 2002). Correspondingly, the interaction terms between climate and growth form in the spatial linear models explaining the frequencies of different sexual systems were significant in most cases. The differences in the effects of climate on proportions of sexual systems between woody and herbaceous growth forms may be partly due to the coevolution of sexual systems with growth forms (Renner and Ricklefs, 1995; Vamosi et al., 2003; Case and Barrett, 2004). Moreover, our results further corroborate previous findings that climate may have influenced the spatial pattern of sexual systems *via* its effect on the composition of plant growth forms (Wang et al., 2020).

We also found that the sexual system composition of woody species was more closely associated with contemporary climate than that of herbaceous species, which is consistent with previous studies at broad scales (Oberle et al., 2009; Wang et al., 2009). Compared with herbaceous species that can be protected from frost by being annual/biennial or by the production of underground buds, woody plant species typically with longer life cycles and large sizes expose their stems and buds (with reproductive organs) in climate. Thus climate has been found to have significant influences on the spatial pattern of woody species (Currie and Paquin, 1987) compared with herbaceous species. These results suggest that the differences in the effects of climate on geographical patterns in proportions of sexual systems for woody and herbaceous species may also be associated with the differences in ecophysiological strategies related to resource acquisition and utilization between growth forms.

### Effect of Evolutionary Time on Proportions of Sexual Systems

For both woody and all species, the proportion of dioecious species was positively correlated with mean genus age per grid cell, which support our hypothesis that dioecy tends to be maintained in regions with older floras. Similarly, the proportion of woody monoecious species was also positively correlated with mean genus age. These results partly support and expand the hypothesis that dioecy and monoecy are often found to be significantly associated with each other across phylogenies of woody angiosperms (Renner and Ricklefs, 1995). In contrast, the proportions of sexual systems for herbaceous species were weakly correlated with mean genus age (e.g. *Apiaceae*, *Asteraceae*, *Cyperaceae*), which might be due to the higher population turnover rate and faster adaptation to climate of herbaceous species than those of woody species (e.g. *Acer* and *Salicaceae*, Obbard et al., 2006; Smith and Beaulieu, 2009). Moreover, the effect of genus age on the proportions of woody sexual systems was much stronger than on those of herbaceous species. The differences in the effect of evolutionary age on proportions of sexual systems between woody and herbaceous growth forms might reflect their differences in evolutionary rates (i.e. speciation and extinction rates). Molecular studies have revealed that compared with herbaceous species, woody species such as trees tend to have longer generation times and lower evolutionary rate (Smith and Beaulieu, 2009), and thus may have lower chances to transit between sexual systems in response to environmental changes.

## Conclusions

Here we mapped the geographical distributions of the frequencies of sexual systems and compared the effects of abiotic and biotic factors on sexual system frequencies for both woody and herbaceous flowering plant species in China. The results suggest that woody and herbaceous species differed significantly in their patterns in the frequencies of sexual systems, and also in the determinants underlying these patterns. The proportions of sexual systems of woody species were more strongly influenced by climate, evolutionary age, and mature height, whereas those of herbaceous species were less influenced by these factors. These findings shed light on previous hypotheses about the association between sexual systems and growth forms, and further demonstrate the necessity to differentiate woody and herbaceous growth forms when investigating the evolution and ecology of sexual systems. It is noteworthy that sexual systems may also vary intraspecifically across populations of a single species (e.g. *Mercurialis annua*, Dorken et al., 2017). Exploring the intraspecific sexual system variations for species with different growth forms along environmental gradients would further improve our understanding on the drivers of sexual system variations. Our study also suggests that compared with both dioecious and hermaphroditic species, more attention should be paid to monoecious species from both evolutionary and ecological perspectives. This study improves our understanding of the mechanisms underlying geographical patterns of reproductive trait diversity across different growth forms and their responses to climate.

## Data Availability Statement

The raw data supporting the conclusions of this article will be made available by the authors, without undue reservation, to any qualified researcher.

## Author Contributions

ZW and YW conceived the idea and design of the study. YW, TL, AL, YLi, and YLiu organized the database. YW and TL performed the statistical analysis. YW wrote the first draft of the manuscript. ZW has comprehensively revised the article. All authors contributed to the article and approved the submitted version.

## Funding

This work was supported by the National Key Research Development Program of China (#2017YFA0605101; #2018YFA0606104), the Strategic Priority Research Program of Chinese Academy of Sciences (#XDB31000000), and National Natural Science Foundation of China (#31901216, #31988102, #31911530102).

## Conflict of Interest

The authors declare that the research was conducted in the absence of any commercial or financial relationships that could be construed as a potential conflict of interest.
