# Supplementary material for: Spatial Patterns and Drivers of Angiosperm Sexual Systems in China Differ Between Woody and Herbaceous Species
Source: Front Plant Sci. 2020 Aug 11;11:1222. doi: 10.3389/fpls.2020.01222 (PMC7432134; doi:10.3389/fpls.2020.01222)
Supplement: Supplementary file 1 [file Table_1.doc]

Supplementary Materials

**Figure A1. The relationships among mean genus ages per grid cell of different versions of phylogenies** including Lu et al. (2018), Zanne et al. (2014) phylogeny, Smith and Brown’s (2018). When the univariate Ordinary Least-Squares regression (modified t tests were used to correct for the effect of spatial autocorrelation on *p* values) for each relationship is significant (P < 0.05), a black line is drawn and the R^2^ of the regression model is shown on the figure.


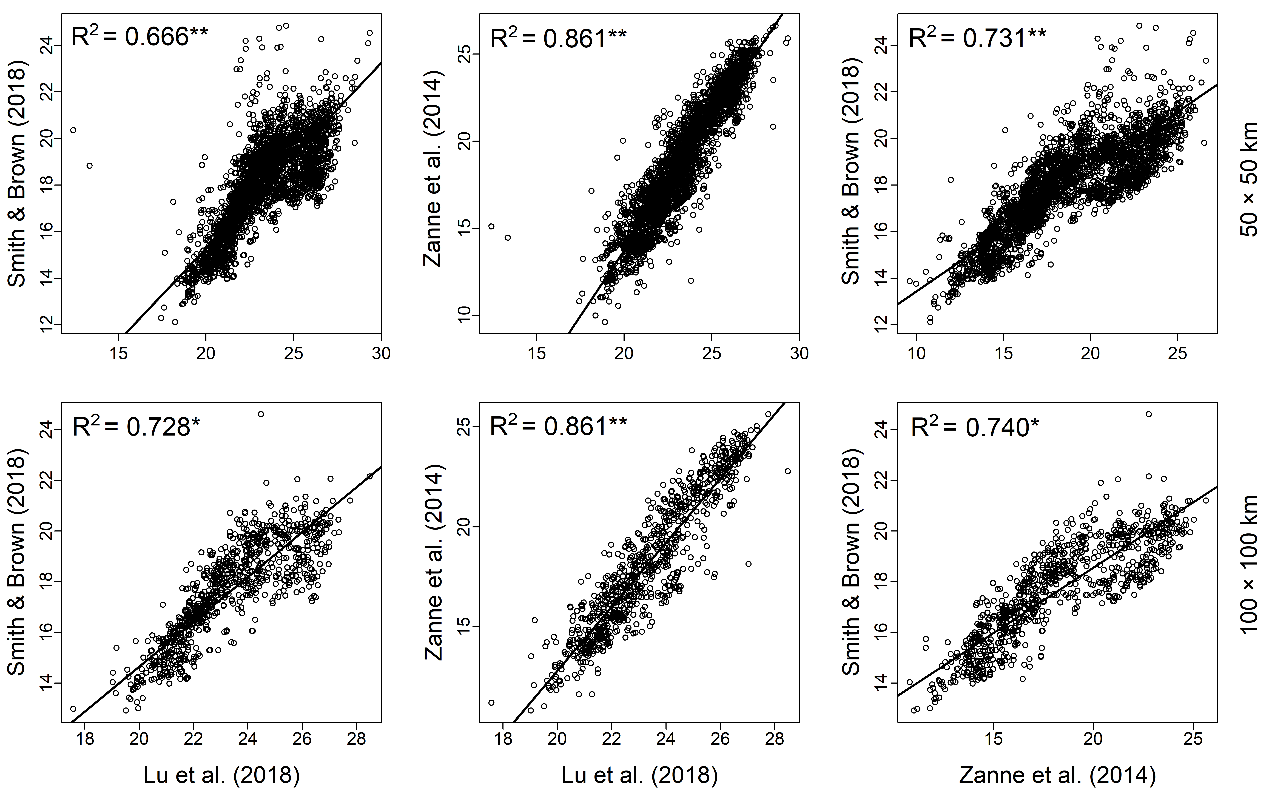


**Table A1 The frequencies of different sexual systems at different taxonomic levels for Chinese angiosperm species. A total of 262 families, 2506 genera, and 19,780 species (including10,202 woody species and 9578 herbaceous species) were included in our analysis. Among these species 2683 are dioecious, 2509 are monoecious, and 14,588 are hermaphroditic species. N = Number of taxa**

| Taxa | Sexual system | Family | | Genus | | Species | |
| --- | --- | --- | --- | --- | --- | --- | --- |
|  |  | N | Prop. (%) | N | Prop. (%) | N. | Prop. (%) |
| All | Dioecy | 97 | 37.0 | 285 | 11.4 | 2683 | 13.6 |
|  | Monoecy | 72 | 27.5 | 348 | 13.9 | 2509 | 12.7 |
|  | Herma. | 211 | 80.5 | 1998 | 79.7 | 14588 | 73.8 |
| Wood | Dioecy | 73 | 46.5 | 206 | 19.0 | 2193 | 21.0 |
|  | Monoecy | 43 | 27.4 | 160 | 14.8 | 1043 | 10.0 |
|  | Herma. | 127 | 80.9 | 779 | 72.0 | 7213 | 69.0 |
| Herb | Dioecy | 48 | 2.54 | 109 | 6.6 | 460 | 4.8 |
|  | Monoecy | 47 | 24.9 | 215 | 13.0 | 1489 | 15.5 |
|  | Herma. | 151 | 79.9 | 1397 | 84.5 | 7629 | 79.7 |

**Table A2 Explanatory power of different variables on geographical patterns in proportions of sexual systems across China.** Univariate generalized linear models (GLMs) with quasi-Poisson residuals were conducted for all relationships and the significance of the regressions was tested using Dutilleul’s (1993) modified t-test. Herma. = hermaphroditism. LuAge, ZanneAge, Smith Age, and MeanAge represent the genus age extracted from Lu et al. (2018), Zanne et al. (2014), Smith & Brown (2018), and the average genus age of these three phylogenies. Significance codes: *** *P* < 0.001, ** *P* < 0.01, * *P* < 0.05, ‘.’ *P* < 0.1.

| Variable | All | | | Herb | | | Wood | | |  |
| --- | --- | --- | --- | --- | --- | --- | --- | --- | --- | --- |
|  | Herma. | Dioecy | Monoecy | Herma. | Dioecy | Monoecy | Herma. | Dioecy | Monoecy |  |
| MTCQ | 0.456. | **0.599 *** | 0.0039 | 0.183. | 0.326. | 0.017 ns | -0.00089 ns | 0.061 ns | 0.163 |  |
| MPWQ(log) | **0.562*** | 0.553. | 0.027 | 0.175. | 0.199 ns | 0.037 ns | **0.247 *** | 0.100 . | **0.291 *** |  |
| AET(log) | **0.564** | 0.573. | 0.019 ns | 0.153 ns | 0.573. | 0.019 ns | **0.247 *** | 0.573. | 0.019 ns |  |
| AI | **0.311*** | **0.393*** | 0.000063 ns | **0.132 *** | **0.238 *** | 0.011 ns | 0.015 ns | -0.00090 ns | 0.088 ns |  |
| LuAge | **0.695*** | **0.737*** | 0.013 ns | 0.174. | 0.157 ns | 0.050 ns | **0.144 *** | 0.037 ns | **0.243 **** |  |
| ZanneAge | **0.746*** | **0.865**** | 0.00025 ns | **0.263 *** | 0.160 ns | 0.106 ns | **0.086 **** | **0.089 ***** | 0.015 ns |  |
| SmithAge | **0.547*** | **0.627*** | 0.00054 ns | 0.124 ns | 0.073 ns | 0.051 ns | **0.213***** | **0.319***** | -0.00017 ns |  |
| MeanAge | **0.739 *** | **0.844 **** | 0.0016 ns | **0.214 *** | 0.173 ns | 0.068 ns | **0.343***** | **0.317***** | **0.0913**** |  |
| Height | **0.773*** | **0.868**** | 0.0037 ns | **0.258 *** | 0.173 ns | 0.097 . | 0.138 ns | 0.032 ns | **0.253.** |  |

**Table A3.1 The slopes of growth forms (woody vs. herbaceous), climate, genus age, plant height and their interaction on proportions of sexual systems** (i.e. dioecy, monoecy, and hermaphroditism) **evaluated using spatial linear models (SLMs) with simultaneous autoregressive errors (SAR).** All continuous predictors were standardized prior analysis. The regression coefficients (slopes) with p < 0.05 is shown in bold. AET = actual evapotranspiration, AI = aridity index, MPWQ = mean precipitation of warmest quarter, MTCQ = mean temperature of coldest quarter, Age = evolutionary age calculated using the phylogeny constructed by Zanne et al. (2014).

| Variables | Dioecy | Monoecy | Hermaphroditism |
| --- | --- | --- | --- |
| Growth Form | **0.165** | **-0.149** | -0.0218 |
| Height | -0.0279 | **0.0895** | -0.0529 |
| AET | 0.00250 | 0.00213 | -0.00497 |
| AI | 0.00336 | -0.000953 | -0.00229 |
| MTCQ | **0.00601** | **-0.00643** | 0.0000974 |
| MPWQ | -0.00274 | 0.00105 | 0.00155 |
| Age | 0.00606 | 0.00599 | -0.0121 |
| Growth Form: AET | **0.0172** | **0.0106** | **-0.0267** |
| Growth Form: AI | **-0.0141** | **-0.00777** | **0.0221** |
| Growth Form: MTCQ | **-0.0499** | **0.00581** | **0.0443** |
| Growth Form: MPWQ | **0.0297** | **0.0157** | **-0.0460** |
| Growth Form: age | **0.0500** | 0.00232 | **-0.0547** |
| Growth Form: height | 0.0309 | **-0.0954** | 0.0554 |
| R^2^ | 0.917 | 0.781 | 0.818 |

**Table A3.2** **The slopes of growth forms (woody vs. herbaceous), climate, genus age, plant height and their interaction on proportions of sexual systems** (i.e. dioecy, monoecy, and hermaphroditism) **evaluated using spatial linear models (SLMs) with simultaneous autoregressive errors (SAR).** All continuous predictors were standardized prior analysis. The regression coefficients (slopes) with p < 0.05 is shown in bold. AET = actual evapotranspiration, AI = aridity index, MPWQ = mean precipitation of warmest quarter, MTCQ = mean temperature of coldest quarter. Genus age calculated using the phylogeny constructed by Lu et al. (2018).

| Variables | Dioecy | Monoecy | Hermaphroditism |
| --- | --- | --- | --- |
| Growth Form | **0.186** | **-0.141** | **-0.0956** |
| Height | -0.00596 | **0.0891** | **-0.0331** |
| AET | **-0.00422** | **0.0113** | -0.00402 |
| AI | **0.00740** | **0.00278** | **-0.0122** |
| MTCQ | **0.00685** | **-0.00896** | **-0.00627** |
| MPWQ | -0.00423 | -0.00234 | **0.0108** |
| Genus Age | **0.00858** | **-0.00749** | 0.000177 |
| Growth Form: AET | **0.0326** | **0.00901** | **-0.0432** |
| Growth Form: AI | 0.00239 | 0.00237 | -0.00393 |
| Growth Form: MTCQ | **-0.0531** | **0.00417** | **0.0513** |
| Growth Form: MPWQ | -0.00294 | **-0.00496** | 0.00779 |
| Growth Form: age | **-0.00400** | **0.0156** | **-0.0125** |
| Growth Form: height | **0.0293** | **-0.0805** | 0.00805 |
| R^2^ | 0.884 | 0.630 | 0.769 |

**Table A3.3** **The slopes of growth forms (woody vs. herbaceous), climate, genus age, plant height and their interaction on proportions of sexual systems** (i.e. dioecy, monoecy, and hermaphroditism) **evaluated using spatial linear models (SLMs) with simultaneous autoregressive errors (SAR).** All continuous predictors were standardized prior analysis. The regression coefficients (slopes) with p < 0.05 is shown in bold. AET = actual evapotranspiration, AI = aridity index, MPWQ = mean precipitation of warmest quarter, MTCQ = mean temperature of coldest quarter. Genus age calculated using the phylogeny constructed by Smith and Brown’s (2018).

| Variables | Dioecy | Monoecy | Hermaphroditism |
| --- | --- | --- | --- |
| Growth Form | **0.125** | **-0.160** | 0.0312 |
| Height | -0.0231 | **0.0913** | **-0.0631** |
| AET | 0.00400 | **0.00641** | **-0.0110** |
| AI | **0.00342** | -0.00126 | -0.00204 |
| MTCQ | **0.00548** | **-0.00791** | 0.00241 |
| MPWQ | -0.00254 | 0.00196 | 0.000393 |
| Age | -0.000203 | **-0.0123** | 0.0144 |
| Growth Form: AET | -0.00550 | -0.00524 | **0.0122** |
| Growth Form: AI | -0.00403 | **-0.00656** | **0.0102** |
| Growth Form: MTCQ | **-0.0115** | **0.0230** | **-0.0121** |
| Growth Form: MPWQ | **0.0138** | **0.0146** | **-0.0279** |
| Growth Form: age | **0.0698** | **0.0396** | **-0.112** |
| Growth Form: height | **0.0632** | **-0.0857** | 0.0157 |
| R^2^ | 0.934 | 0.804 | 0.879 |

**Table A4.1 The slopes and significance of different predictors on proportions of sexual systems evaluated using spatial linear models (SLMs) with simultaneous autoregressive errors (SAR).** The regression coefficients (slopes) with p < 0.05 is shown in bold. Here genus age was extracted from the phylogeny constructed by Zanne et al. (2014).

| Variable | All | | | Herb | | | Wood | | |
| --- | --- | --- | --- | --- | --- | --- | --- | --- | --- |
|  | Herma. | Dioecy | Monoecy | Herma. | Dioecy | Monoecy | Herma. | Dioecy | Monoecy |
| AET | **-0.0179** | **0.00988** | **0.00797** | -0.00353 | 0.00162 | 0.00191 | **-0.0502** | **0.0350** | **0.0152** |
| AI | -0.00132 | **0.00470** | **-0.00338** | -0.00159 | **0.00371** | -0.00212 | **0.0202** | **-0.0119** | **-0.00822** |
| MTCQ | **0.00808** | -0.000473 | **-0.00762** | 0.00139 | **0.00390** | **-0.00529** | **0.0305** | **-0.0334** | **0.00296** |
| MPWQ | -0.000108 | **-0.00590** | **0.00602** | 0.000537 | **-0.00296** | 0.00242 | **-0.0457** | **0.0294** | **0.0162** |
| Age | **-0.00853** | **0.0154** | **-0.00693** | -0.00267 | 0.000220 | 0.00245 | **-0.0239** | **0.0197** | **0.00422** |
| Height | **-0.0197** | **0.0158** | 0.00391 | **-0.00760** | 0.001997 | **0.00560** | **-0.0344** | **0.0255** | **-0.00892** |
| R^2^ | 0.943 | 0.922 | 0.706 | 0.320 | 0.432 | 0.691 | 0.911 | 0.938 | 0.611 |

**Table A4.2** **The slopes and significance of different predictors on proportions of sexual systems (50 × 50 km) evaluated using** **spatial linear models (SLMs) with simultaneous autoregressive errors (SAR).** The regression coefficients (slopes) with p < 0.05 is shown in bold. Here genus age was extracted from the phylogeny constructed by Lu et al. (2018).

| Variable | All | | | Herb | | | Wood | | |
| --- | --- | --- | --- | --- | --- | --- | --- | --- | --- |
|  | Herma. | Dioecy | Monoecy | Herma. | Dioecy | Monoecy | Herma. | Dioecy | Monoecy |
| AET | **-0.0161** | -0.00188 | **0.0180** | **-0.00615** | **-0.0031** | **0.0118** | **-0.0482** | **0.0274** | **0.0209** |
| AI | **-0.0140** | **0.0145** | -0.000533 | **-0.0118** | **0.00957** | **0.00222** | **-0.0159** | **0.0113** | **0.00454** |
| MTCQ | **0.00851** | 0.000660 | **-0.00917** | **-0.00244** | **0.00880** | **-0.00635** | **0.0479** | **-0.0444** | **-0.00348** |
| MPWQ | **0.0139** | **-0.0133** | -0.000623 | **0.0102** | **-0.00705** | **-0.00314** | **0.0181** | **-0.00943** | **-0.00871** |
| Age | 0.00103 | **0.00573** | **-0.00677** | 0.000537 | **0.00540** | **-0.00593** | **-0.0130** | **0.00476** | **0.00825** |
| Height | **-0.0371** | **0.0377** | -0.000598 | **-0.00517** | -0.000324 | **0.00550** | **-0.0181** | **0.0147** | **0.00333** |
| R^2^ | 0.807 | 0.888 | 0.179 | 0.250 | 0.478 | 0.259 | 0.875 | 0.779 | 0.411 |

**Table A4.3 The slopes and significance of different predictors on proportions of sexual systems evaluated using spatial linear models (SLMs) with simultaneous autoregressive errors (SAR).** The regression coefficients (slopes) with p < 0.05 is shown in bold. Here genus age was extracted from the phylogeny constructed by Smith and Brown (2018).

| Variable | All | | | Herb | | | Wood | | |  |
| --- | --- | --- | --- | --- | --- | --- | --- | --- | --- | --- |
|  | Herma. | Dioecy | Monoecy | Herma. | Dioecy | Monoecy | Herma. | Dioecy | Monoecy |  |
| AET | **-0.0191** | **0.0120** | **0.0115** | **-0.0105** | 0.00387 | **0.00658** | **-0.0140** | **0.0100** | 0.00290 |  |
| AI | -0.00339 | **0.00848** | -0.00168 | **-0.00240** | **0.00249** | -0.0000768 | -0.000801 | **0.00500** | **-0.00494** |  |
| MTCQ | **0.00901** | **0.00521** | **-0.00571** | 0.000626 | **0.00225** | **-0.00280** | **-0.0191** | 0.00248 | **0.0162** |  |
| MPWQ | 0.00283 | **-0.0167** | 0.00122 | 0.00110 | -0.000363 | -0.000772 | -0.000989 | -0.00988 | **0.0130** |  |
| Age | -0.000575 | **0.00551** | **-0.00947** | **0.00471** | **0.00203** | **-0.00676** | **-0.0619** | **0.0447** | **0.0170** |  |
| Height | **-0.0273** | **0.0270** | 0.00118 | **-0.00490** | -0.000992 | **0.00585** | **-0.0307** | **0.0267** | **0.00362** |  |
| R^2^ | 0.830 | 0.904 | 0.324 | 0.333 | 0.453 | 0.239 | 0.669 | 0.568 | 0.530 |  |
